# Adsorption of K Ions on Single-Layer GeC for Potential Anode of K Ion Batteries

**DOI:** 10.3390/nano11081900

**Published:** 2021-07-24

**Authors:** Yue Ma, Sen Xu, Xiaofeng Fan, David J. Singh, Weitao Zheng

**Affiliations:** 1 Key Laboratory of Automobile Materials of MOE and College of Materials Science and Engineering, Jilin University, Changchun 130012, China; may19@mails.jlu.edu.cn (Y.M.); xusen16@mails.jlu.edu.cn (S.X.); wtzheng@jlu.edu.cn (W.Z.); 2 Department of Physics and Astronomy, University of Missouri, Columbia, MO 65211, USA; singhdj@missouri.edu; 3 Department of Chemistry, University of Missouri, Columbia, MO 65211, USA; 4 State Key Laboratory of Automotive Simulation and Control, Jilin University, Changchun 130012, China

**Keywords:** potassium ion batteries, anode, g-GeC, first-principle methods

## Abstract

Potassium ion batteries (KIBs) are considered as promising alternatives to lithium ion batteries (LIBs), following the rapid increase of demand for portable devices, and the development of electric vehicles and smart grids. Though there has been a promising breakthrough in KIB tech niques, exploring the promising anode materials for KIBs is still a challenge. Rational design with first-principle methods can help to speed up the discovery of potential anodes for KIBs. With density functional calculations, GeC with graphene-like 2D structure (g-GeC) is shown to be a desired anode material for applications in KIBs. The results show that the 2D g-GeC with a high concentration of K ions is thermodynamically stable, due to the strong interaction between C and Ge in GeC layer with the proper interaction between K and GeC. The storage capacity can be about 320 mAh/g, higher than that (279 mAh/g) in graphite. The low energy barrier (0.13 eV) of K ions diffusion on the honeycomb structure with proper voltage profile indicates the fast charge transfer. These theoretical finds are expected to stimulate the future experimental works in KIBs.

## 1. Introduction

As the most commonly used commercial electrochemical batteries, lithium-ion batteries (LIBs) have been used as excellent energy storage systems to power various electronic products in smart electronics and have potential applications in the electric vehicle market since their commercialization [1,2,3]. However, with the miniaturization and lightening of equipment and people’s control of cost prices, LIBs’ techniques are being restricted by various factors, such as safety issues and scarcity of raw materials [4,5,6]. At present, the frequency of use of LIBs continues to increase, while the contradiction of raw materials faced by LIBs is increasingly exposed. Thus, people are gradually turning their attention to other metal-ion batteries [7,8,9]. Since potassium and lithium are of the same main group, the standard electrode potential of potassium-ion batteries (KIBs) is very close to that of LIBs. In addition, KIBs have higher ionic conductivity and less complex interfacial reaction than sodium-ion batteries as alternative potential techniques [10,11,12]. On the other hand, potassium is rich in reserves, which can reduce production costs for mass produce. In this way, KIBs have gradually aroused people’s enthusiasm for research in recent years [13].

KIB mainly consists of anode, cathode and electrolyte, in which the electrode materials determine the capacity and stability of the batteries. Due to the large ion radius of potassium ions and the improper interaction between traditional anode materials and K ions, the instability of anode materials is usually caused [10]. Therefore, looking for higher performance anode material has become one of the urgent tasks for the further development of KIBs.

For the past few years, two-dimensional materials with large surface areas that can achieve rapid ion diffusion have attracted great interest in the field of rechargeable ion batteries [9,14,15]. Graphene, the first and most fascinating 2D material, could be successfully synthesized by different methods, such as mechanical exfoliation of pristine graphite, oxidation-reduction method, chemical vapor precipitation and epitaxial growth method. It has been proven that graphene and graphene-based nanostructures can be used as an ideal negative electrode material for rechargeable ion batteries, such as LIBs [16,17,18,19]. Similarly, germanene and silicene as ion battery anode materials have been studied [20,21]. The conductivity of germanium without doping or alloying is not good, and germanium and carbon composite material has been proposed as a potential anode material with good conductivity due to sp^2^-carbon being in composite [22,23,24,25]. Recently, a graphene-like Ge/C composite called germagraphene (g-GeC) has been proposed, predicted by first-principles calculations to be a two-dimensional nonmagnetic semiconductor with a direct band gap of about 2.1 eV at K (K’) points [26,27,28,29,30]. The structure is the same as germanene, silicene and other honeycomb-like structures, but different from germanium is that it is a plane structure without buckling height [29]. Khossossi et al. found that GeC can be used as the anode material for lithium-sodium ion batteries [31], and proved that the redistribution of the charge in the germanium-benzene monolayer could give rise to the stronger adsorption of Li/Na ions on GeC than on the original graphene material.

In this paper, the first principles method was used to analyze the structure and electronic properties of g-GeC with the adsorption of potassium ions. The unique structure of g-GeC makes the germanium atom have an appropriate synergistic stabilization effect on the potassium ion, which makes the potassium ion more easily adsorbed on the germanium atom, different from Li and Na ions’ adsorption on g-GeC as observed by Khossossi et al. [31]. The adsorption structure is very stable due to the larger adsorption strength, compared to the binding energy of K in bulk K. By considering the different adsorption processes with different contents of K on g-GeC, we predicted the maximum capacity of GeC as the anode for KIBs with proper open-circuit voltage. By analyzing the ion diffusion properties, the potential barrier of the potassium ion diffusion on g-GeC is found to be low, especially at high content of K. The high migration rate of potassium ions with proper conductivity on g-GeC meets the requirement of the mobility of an anode. By analyzing the electronic properties, such as band structure, density of states and charge distribution, it is systematically proved that GeC has the potential to be used as anode material for KIBs.

## 2. Materials and Methods

The structure of GeC involved in this paper shows hexagonal symmetry similar to graphene, where the germanium and the carbon atoms are arranged in alternating sp^2^-hybridization to form a network. Its stable two-dimensional (2D) geometry is planar, similar to graphene, with vertical electron orbitals for π bonds. The dynamic stability of the structure of GeC has been demonstrated [29,31,32].

All our first-principles calculations were performed on the basis of density functional theory as implemented in Vienna Ab initio Simulation Package (VASP) [33,34]. The generalized gradient approximation (GGA) as formulated and parameterized by the Perdew –Burke–Ernzerhof (PBE) functional was used for the electron–electron exchange correlation [35]. Electron−ion interactions were described by the method of projector augmented wave (PAW) [36,37]. An energy cutoff of 550 eV was selected for the plane wave expansion of valence electron wave functions. The effect of van der Waals (vdW) interaction was taken into account by using the DFT-D3 approach [38]. The Brillouin zones were sampled using a reciprocal spacing of 0.02 Å^−1^, and the Gamma-centered method was followed. The vacuum of 20 Å was applied along the direction perpendicular to avoid the virtual interactions along *z* direction due to the influence of periodic boundary conditions.

## 3. Results and Discussion

### 3.1. Adsorption of K Ions on GeC in Different Supercell

**Figure 1:**
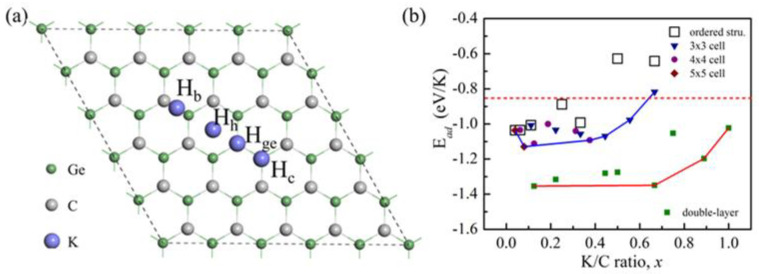
(**a**) Four possible K ion adsorption sites in a 5 × 5 × 1 supercell of g-GeC surface, (**b**) the adsorption energies of K ions on g-GeC as functions of the ratio of K/C in various supercells for both one-surface and two-surface adsorption. Note the green square in (**b**) indicates the two-surface adsorption of K ions on g-GeC (double-layer model).

We began with the search for proper absorption sites at a low concentration of K (K/C ratio *x* = 0.04). The calculations were performed in a 5 × 5 × 1 supercell of g-GeC, as depicted in Figure 1a. Four possible adsorption sites with high symmetry were under our consideration, including H_ge_, H_c_, H_h_ and H_b_, corresponding K ions’ adsorption on the Ge atom, C atom, central position of hexagonal ring and bridge site between Ge and C atoms, respectively. The stable existence of potassium ions in the structure can be determined by the binding between adatom and structure. To evaluate the binding strength, the adsorption energy can be analyzed by the following formula [19]:

(1) $$E_{ad}=(E_{K-GeC}-\;E_{GeC}-E_{K})/n$$

where *E_K-GeC_*, *E_GeC_* and *E_K_* are total energies of GeC with K adsorption, isolated GeC and isolated K atom, respectively, and *n* is the number of adsorbed K atoms on GeC in the adsorption state. According to this definition, negative adsorption energy represents a stable configuration with favorable interaction, and positive adsorption energy represents an unstable structure. The adsorption energies of different adsorption sites can be obtained in Table 1. It is clear that there is proper interaction between K and GeC. H_ge_ is the most stable absorption site, followed by H_h_ and H_c_. In the optimization process, if the atomic position in the GeC plane isn’t fixed, the potassium atom at the bridge position is accustomed to migrating over the Ge atom. Here, it is confirmed that the bridge site is unstable.

With the K ions’ adsorption at H_ge_ sites, we analyzed the change of adsorption energy following the increase of K content. By considering the ordered adsorption state of K ions, we analyzed the change of K concentration from *x* = 0.04 to 0.667 with the construction of different models, including the adsorption of one K ion in 5 × 5, 4 × 4, 3 × 3, 2 × 2, √3 × √3 and √2 × √2 supercells and two K ions in a √3 × √3 supercell. The typical adsorption configurations of a K ion in high content are shown in Figure S1 of Supplementary Materials. The corresponding adsorption energies are shown with the hollow squares in Figure 1b. It is clear that there is a decreasing trend of adsorption strength with the increase of K content. The binding energy of K atom in bulk K is about 0.87 eV. This means that if we use the bulk K as the reference state, as the red dot line shows in Figure 1b, it is possible that the adsorption becomes improper in the electrochemical environment if the concentration is more than 0.5.

We have noticed that the interaction between K ions on g-GeC is repulsive, while the interaction between K and the Ge atom under the K ion induces a local strain. It is possible that the energies of other configurations are lower than that of the structure with the ordered adsorption state of K at the same concentration. We constructed the other possible configurations of K adsorption with different contents of K in which the distance between K ions on g-GeC is as large as possible in the restriction of periodic boundary condition. These configurations are constructed in supercells 3 × 3, 4 × 4, and 5 × 5, as shown in Figures S2–S4 of Supplementary Materials. The results are shown in Figure 1b. Indeed, it is observed that there are other low energy configurations. With the consideration of these configurations, the maximal concentration of K adsorption is about 0.67.

As the concentration increases, K ions deviate from the adsorption sites of Ge due to the Coulomb repulsion between K ions, as shown in Figures S2 and S3. It is possible to reduce the repulsive interaction between K ions to a large extent if K ions are adsorbed by means of two-surface adsorption on g-GeC layer (double-layer model). We constructed the configurations with different contents of K by proposing the K adsorption in different supercells of √3 × √3, 2 × 2, 3 × 3 and 4 × 4 in Figures S5–S8 of Supplementary Materials. In these configurations, the K ions are adsorbed on both surfaces in the stagger way to reduce the local strain. It is known that if the adsorption strength (|*E_ad_*|) per K ion on g-GeC is larger than the binding energy of a K atom in bulk K, there will be no potassium clusters formed in the process of K loading [19]. In Figure 1b, it is clear that the adsorption with two surfaces is more stable than that just on one surface. The maximal concentration of K adsorption can reach *x* = 1. In Figure S9, the change of lattice parameter as a function of K concentration is plotted for both adsorption processes. The change in the lattice constant is less noticeable as the concentration of K increases. Thus, the strain in the entire lattice plane due to K adsorption is weak.

In order to check the stability of this material system with K adsorption, we calculated the formation energy due to the K adsorption with the formula:

(2) $$E_{f}=(E_{K-GeC}-x\;E_{K-bulk}-E_{GeC}\;)/(1+x)$$

where *E_K-GeC_, E_GeC_* and *E_K-bulk_* are total energies of GeC with K adsorption, isolated GeC and bulk K, respectively. The results for single-layer K adsorption and double-layer adsorption (two sides of g-GeC) are shown in Figure S10. It is clear that this material with K adsorption is energetically stable. It is also noticed that in other materials, such as single layer Mxene, it is possible that double-layer K ions are adsorbed by way of one side adsorption [39]. In the single-layer g-GeC, the adsorption of K on g-GeC is not so strong that there is a double-layer of K ions adsorption on one side.

**Figure 2:**
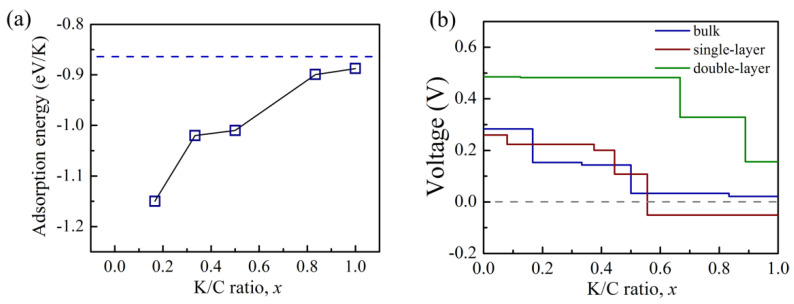
(**a**) The adsorption energy as a function of K concentration for bulk g-GeC and (**b**) the voltage profile of open-circuit voltage for K interaction in bulk g-GeC, one side adsorption (single-layer model) and two-surface adsorption (double-layer model) on monolayer g-GeC.

We also analyzed the intercalation of K ions in bulk g-GeC in order to estimate the adsorption in multi-layer g-GeC. The adsorption models for different concentrations of K are depicted in Figures S11 and S12. As shown in Figure 2a, it was found that the maximal concentration was also *x* = 1. The theoretical capacity is approximately evaluated with the maximal concentration of K ions in the K loading process. It can be calculated by the formula *C* = *xF/M_GeC_*, where *F*, *x*, and *M_GeC_* are Faraday constant (26.8 Ah mol^−1^), K concentration inserted in g-GeC per formula cell, and mole mass of one g-GeC formula cell with the inserted K, respectively. It is clear that when potassium performs two-surface adsorption on g-GeC monolayer or intercalates into bulk g-GeC, the capacity at the insertion concentration of *x* = 1 is 316.7 mA h g^−1^.

As mentioned above, we checked the adsorption of K ions on mono-layer g-GeC and in bulk g-GeC. In the reasonable range of adsorption concentrations, the K ions can be stably adsorbed on g-GeC. For the anode applications, we can evaluate the open-circuit voltage (OCV) with the K loading by the voltage profile. It is related to the Gibbs free energies in both charged and discharged states by *V* = −Δ*G/*Δ*xF*, where Δ*G* and Δ*x* are the change of Gibbs free energies and K concentration in both states, respectively. In the intercalation of ions, such as Li, Na and K in anode, the contributions of vibrational and configurational entropy are small and can be ignored. Thus, the difference in Gibbs energies can be approximated to the change of energy by Δ*G ≈ E*(K*_x_*GeC) − *E*(GeC) − *xE*(K). In Figure 2b, open circuit voltage curves are plotted for the single-layer adsorption on one side of g-GeC monolayer, double-layer adsorption on two sides of g-GeC monolayer and intercalation of K in bulk g-GeC. From the K concentration of *x* = 1 with two-surface adsorption in K-loading state, the OCV is about 0.15 V. In the single-layer adsorption model, the OCV becomes negative when the K concentration is more than 0.55. For the intercaltion of K in bulk g-GeC, there is a good voltage profile, with the largest OCV less than 0.3 V. Generally speaking, materials with an averaged OCV lower than 1 V can be used as the anode in KIBs. Thus, the applicable OCV (the largest OCV less than 0.5 V) provides feasibility for the GeC to be applied as anodes for KIBs.

### 3.2. Analysis of Electronic Properties

**Figure 3:**
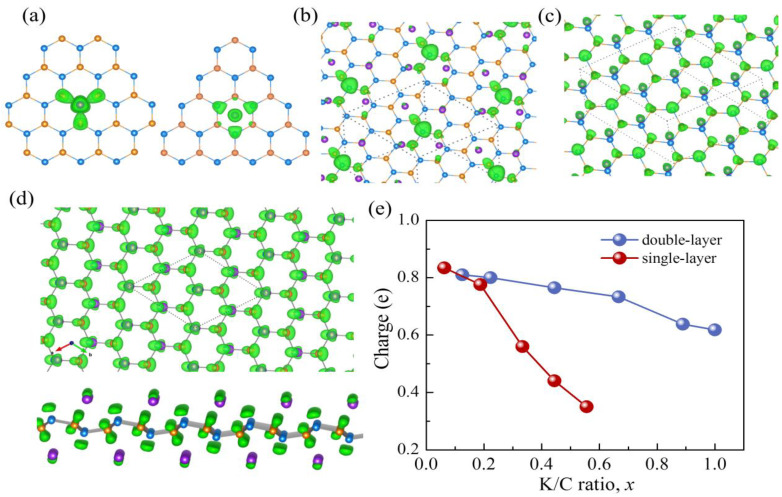
Distribution of electron charge density difference at the K/C ratio of (**a**) *x* = 0.0625 with K adsorption on H_ge_ and H_h_ sites in 4 × 4 × 1 supercell, (**b**) *x* = 0.44 and (**c**) *x* = 0.67 in 3 × 3 × 1 supercell, and (**d**) *x* = 1 with K adsorption by way of two-surface adsorption in 2 × 2 × 1 supercell, (**e**) the change of averaged charge amount that K loses with increase of K concentration. The orange, blue, and purple spheres represent C, Ge and K atoms, respectively. The green is for the transferred charge from K to g-GeC.

To gain a more detailed insight into the mechanism of the diverse adsorption behavior of K ions on the GeC, charge density difference is applied for describing the charge distribution, and the charge density difference in real space can be estimated by the following equation [40]:

(3) $$\Delta CHG(r)=CHG_{K-GeC}(r)-CHG_{GeC}(r)-CHG_{K}(r)$$

where *CHG_K-GeC_*(*r*), *CHG_GeC_*(*r*) and *CHG_K_*(*r*) are the electronic charge distributions of the K-adsorbed g-GeC, free g-GeC and isolated potassium atom, respectively. In Figure 3a, potassium atoms are adsorbed at H_Ge_ and H_h_ sites on the left and right sides, respectively. The charge redistributions in the K adsorption configurations are rather symmetrical.

**Figure 4:**
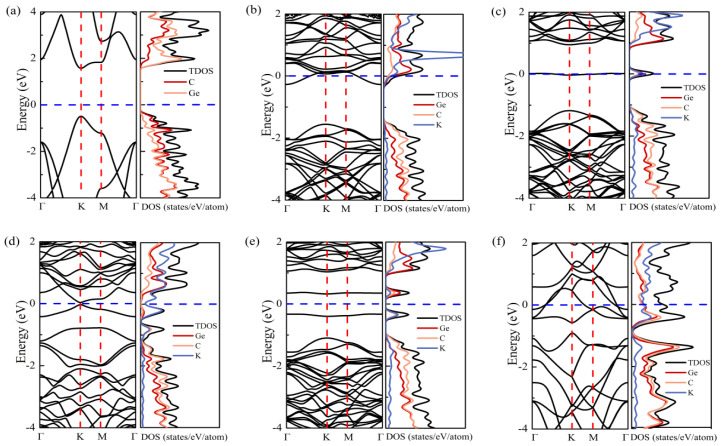
Band structure, density of states (DOS) and partial density of states (PDOS) of (**a**) pristine g-GeC, (**b**,**c**) g-GeC with K adsorption on H_h_ and H_ge_ sites at low concentration of *x* = 0.0626, (**d**) g-GeC with K concentration of *x* = 0.44, and (**e**,**f**) g-GeC with K adsorption by way of two-surface adsorption with K concentrations of *x* = 0.125 and *x* = 1.

The K ion at H_h_ site has a uniform charge transfer mainly with the surrounding three carbon atoms. In Figure 4a, we calculated the band structure and partial density of states (PDOS) of pristine g-GeC. It is clear that g-GeC is a semiconductor, as the previous reports. The states near the valance band’s top are mainly from the contribution of carbon atoms, while the states near the conduction band’s bottom are mainly due to the Ge *p*-orbitals. Therefore, the change of K is transferred to the nearby C atoms and thus, results in the up-shift of Fermi level into conduction bands in Figure 4b, since the energy level of K *s*-orbital is higher than the conduction band’s bottom. For the adsorption of the K ion on H_Ge_ site, the charge of K is transferred to not only the nearby C atoms, but also the nether Ge atom. The interaction between K and the nearby three C atoms results in a down-shift of the nether Ge atom from g-GeC plane. The stable state among the K ion, nearby three C atoms, and nether Ge is formed by the electrostatic interaction with charge transfer. Thus, a localized band appears in the band gap of g-GeC in Figure 4c. It results from the contribution of K and nearby C and Ge atoms. Therefore, the adsorption of the K ion on H_ge_ site is more stable than on H_h_ site in Table 1. This is different from the adsorption of Li and Na with H_h_ site on g-GeC [31].

From the perspective of high-concentration adsorption, we plotted the charge density difference at the concentrations of *x* = 0.44 and 0.67 in Figure 3b,c, respectively. It is clear that the repulsion among K ions results in the deviation of K adsorption on H_ge_ in Figure 3b. The charge transferred from K is localized on a Ge atom with the nearby three C atoms which is in the center of four K ions. The deviation of K on H_ge_ can be ascribed to the electrostatic trap of the aggregated charge on Ge at the center of four K ions. With the increase of K concentration up to *x* = 0.67 in Figure 3c, the arrangement of K ions comes to be ordered on H_ge_ sites with the pattern of hexagonal symmetry. The charge transferred from K is accumulated on a Ge atom with the nearby three C atoms, which are in the center of a six-ring formed by six K ions in Figure 3c. With the increase of K concentration, the localized band in the band gap disappears. As shown in Figure 4d and Figure S13, the band structures of *x* = 0.44 and 0.67 indicate that the charge transfer results in the up-shift of Fermi level. The weak strain of g-GeC induced by the adsorption of K ions results in the closing of the band gap. The system becomes conductive.

Subsequently, we checked the change of electronic structure due to the K adsorption through the process of two-surface adsorption. For example, at *x* = 0.125 with two K atoms adsorbed on g-GeC with both surfaces in a supercell of 4 × 4. In Figure 4e, two K atoms induce two localized bands in the band gap of g-GeC, similar to the adsorption on one surface with the low concentration of K in Figure 4c. Following the increase of K content, the Fermi level is shifted up and the system becomes conductive, as shown in Figure 4f. The charge transfer at *x* = 1 is shown in Figure 3d. The K ions form the structure of up-down chains and the induced weak strain from the K adsorption results in the zigzag structure of the g-GeC plane along a direction perpendicular to the K chain direction, as shown by the side view in Figure 3d. The transferred charge is evenly distributed on both sides of the g-GeC plane.

To qualitatively estimate the charge transfer process, we illustrate the change of transferring charge with the increase of K concentration by the analysis of Bader charge on K in Figure 3e. The amount of charge transferred by each K ion in the two-surface adsorption is greater than that in the one-surface adsorption for each concentration of K. This proves that the interaction between g-GeC and K ions by way of two-surface adsorption is stronger than that with the one-surface adsorption, observed in Figure 1b. With the increase of concentration, the amount of charge transfer in the monolayer visibly decreases. This conforms to the trend of adsorption energy and proves that the adsorption strength of the K ion on g-GeC gradually becomes weak following the increase of K concentration.

### 3.3. Diffusion Mechanism of Potassium Ion

**Figure 5:**
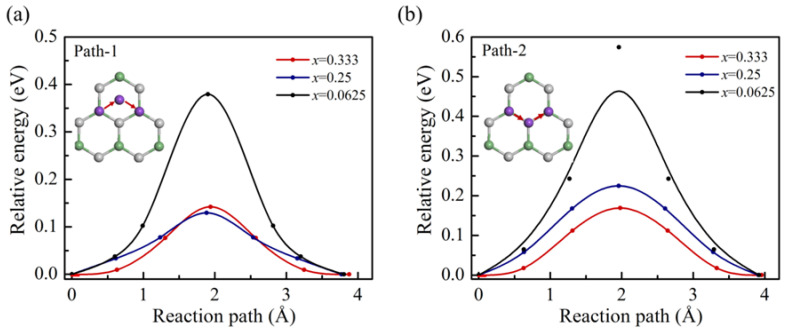
The diffusion barrier profiles of one K ion migration on g-GeC along (**a**) path-1 (Ge-H_h_-Ge) and (**b**) path-2 (Ge-C-Ge) at different concentrations.

The charge and discharge rate capability are mainly determined by the ion migration performance. The nudged elastic band (NEB) method has been used to investigate the diffusion properties at different concentrations. In the course of our calculations, the potassium ion has preferred to stay above the Ge atom, so we chose the positions above the adjacent two Ge atoms as the initial and final state positions. There are two typical paths shown in the insets of Figure 5a,b, respectively. The path-1 is Ge-H_h_-Ge, where H_h_ is the hexagonal site. The path-2 is Ge-C-Ge. Both paths can complete the diffusion in the entire material range and belong to the complete diffusion path. The K ion along both path-1 and path-2 diffuses through H_h_ and H_c_ sites, respectively. It can be seen in Figure 5, compared with path-2, the diffusion energy barrier of path-1 is lower. Thus, the K ions are inclined to diffusion along path-1 through H_h_ site. In this way, there are three symmetric three pathways at each H_ge_ site. Following the K loading, the diffusion barrier decreases for both paths. At the higher concentration, the diffusion barrier becomes similar to path-1 and path-2. At *x* = 0.33, the energy barrier of diffusion is about 0.13 eV. This low diffusion barrier indicates the fast charge/discharge rate for K on g-GeC.

## 4. Conclusions

As mentioned above, we have systematically studied the impact of g-GeC as an anode on the performance of KIBs based on the density functional theory. In the processes, we found that H_Ge_ site on g-GeC is the most favorable adsorption site for a single K ion, and the adsorption energy is appropriate, with a value of −1.04 eV. Because of the enhancement of the repulsion between potassium and potassium, the adsorption strength of each K ion decreases gradually along with the increase of the K/C ratio. The theoretical capacity is estimated to be up to about 320 mA h g^−1^. In addition, g-GeC exhibits metallic properties during the whole potassium insertion process. This indicates that the electrical conductivity of the g-GeC structure is gradually improving with the K loading, since the pristine g-GeC is a semiconductor. In the end, the diffusion potential along the path Ge-H_h_-Ge is about 0.13 eV at the concentration of *x* = 0.33, which meets the requirement of high charging and discharging efficiency of KIBs. In view of the merits above, g-GeC has the potential to be an alternative anode material for KIBs.

## Figures and Tables

**Table 1:** The parameters of K adsorption on g-GeC layer calculated in supercell 5 × 5 × 1, including the distance between K and g-GeC layer and corresponding adsorption energy at different absorption sites.

Site	H_ge_	H_c_	H_h_	H_b_
**Distance (Å)**	2.50	2.88	2.53	
***E_ad_* (eV)**	−1.04	−0.44	−0.57	unstable

## Data Availability

All necessary data have been illustrated in the manuscript and the Supplementary Materials. Additional data may be obtained from corresponding authors with their permission.

## Supplementary Materials

The following are available online at https://www.mdpi.com/article/10.3390/nano11081900/s1. Figures S1–S4: The structure models for K-adsorbed g-GeC in the way of one-surface adsorption with different concentrations of K, Figures S5–S8: The structure models for K-adsorbed g-GeC in the way of two-surface adsorption with different concentrations of K, Figure S9: Lattice changes with the K concentration, Figure S10: formation energies, Figures S11 and S12: The structure models for K-adsorbed bulk g-GeC, Figure S13: Electronic properties of g-GeC with *x* = 0.67.
