# Supplementary material for: Adsorption of K Ions on Single-Layer GeC for Potential Anode of K Ion Batteries
Source: Nanomaterials (Basel). 2021 Jul 24;11(8):1900. doi: 10.3390/nano11081900 (PMC8401946; doi:10.3390/nano11081900)
Supplement: Supplementary file 1 [file nanomaterials-11-01900-s001.zip › nanomaterials-1278280-supplementary.pdf]

Supporting Information

Adsorption of K Ions on Single-layer GeC for Potential Anode of K Ion Batteries

Yue Ma [1], Sen Xu [1], Xiaofeng Fan [1,*], David J. Singh [2,3] and Weitao Zheng [1,4]

[1] Key Laboratory of Automobile Materials of MOE and College of Materials Science and Engineering,
Jilin University, Changchun 130012, China; may19@mails.jlu.edu.cn (Y.M.); xusen16@mails.jlu.edu.cn (S.X.); wtzheng@jlu.edu.cn (W.Z.)

[2] Department of Physics and Astronomy, University of Missouri, Columbia, MO 65211, USA;
singhdj@missouri.edu

[3] Department of Chemistry, University of Missouri, Columbia, MO 65211, USA

[4] State Key Laboratory of Automotive Simulation and Control, Jilin University, Changchun 130012, China

* Correspondence: xffan@jlu.edu.cn; Tel.: +86-159-4301-3494

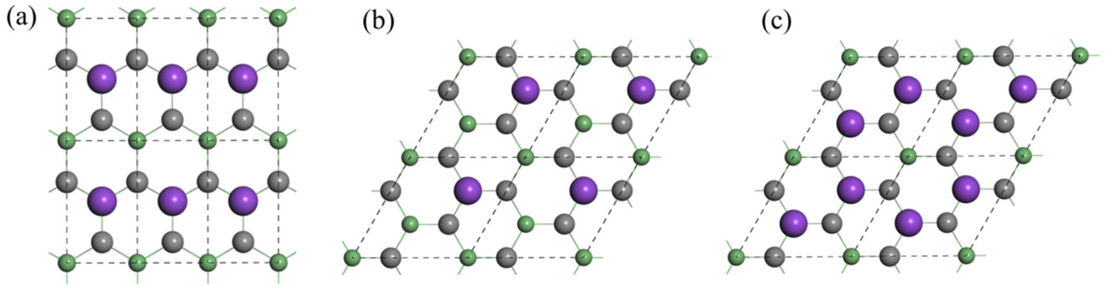

Figure S1. Top view of structures of K-adsorbed of g-GeC with the ratio of K/C of (a) x=0.5 in the √2×√2×1 supercell, (**b**) x= 0.33 and (**c**) x=0.667 in the √3×√3×1 supercell

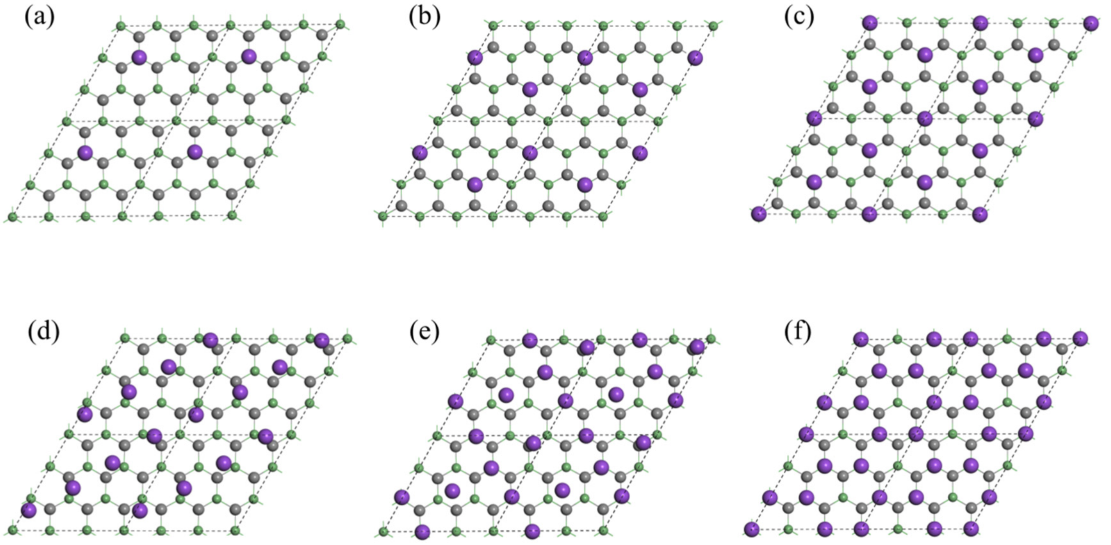

Figure S2. Top view of structures of K-adsorbed g-GeC in 3 × 3 × 1 supercell

with the ratio of K/C of (a) x= 0.11, (**b**) x= 0.22, (**c**) x=0.33, (**d**) x=0.44, (**e**) x=0.56 and (**f**) x=0.67

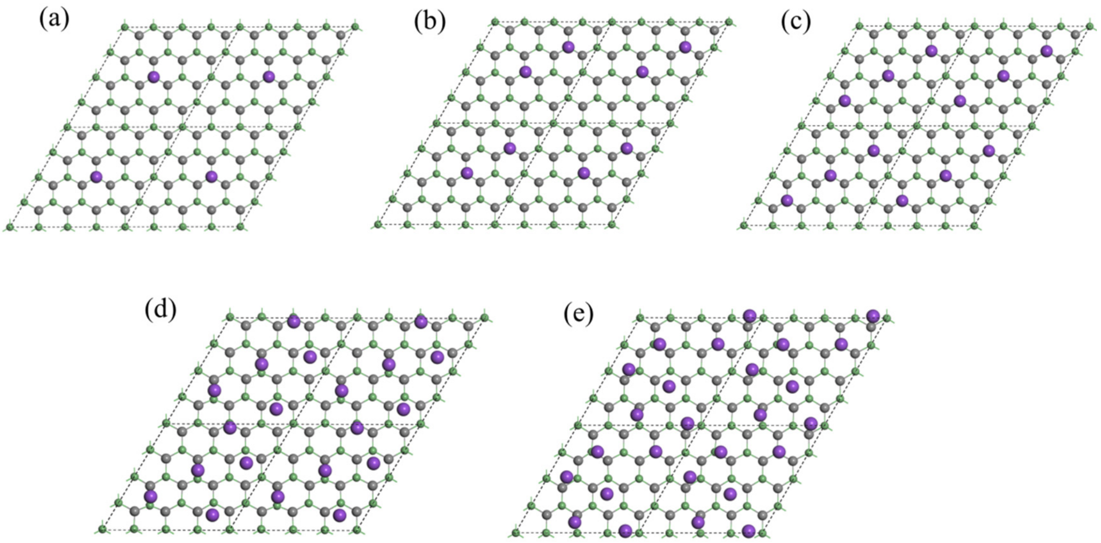

Figure S3. Top view of structures of K-adsorbed g-GeC in 4 × 4 × 1 supercell with the ratio of K/C of (**a**) x=0.0625, (**b**) x=0.125, (**c**) x=0.1875, (**d**) x=0.3125 and (**e**) x=0.375

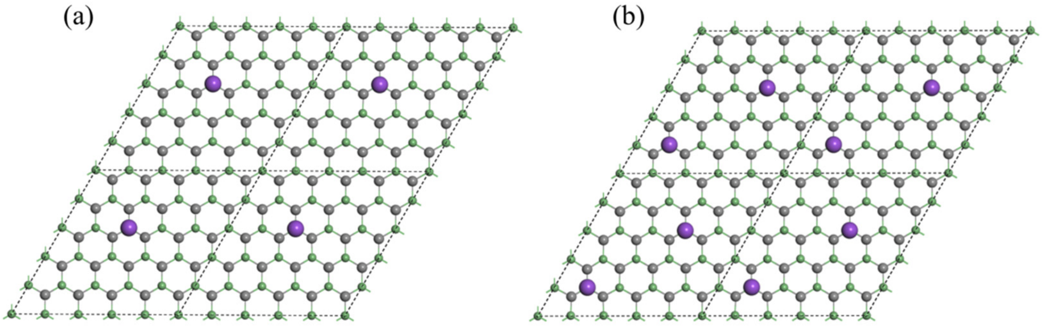

Figure S4. Top view of structures of K-adsorbed g-GeC in 5 × 5 × 1 supercell with the ratio of K/C of (**a**) x=0.04 and (**b**) x=0.08

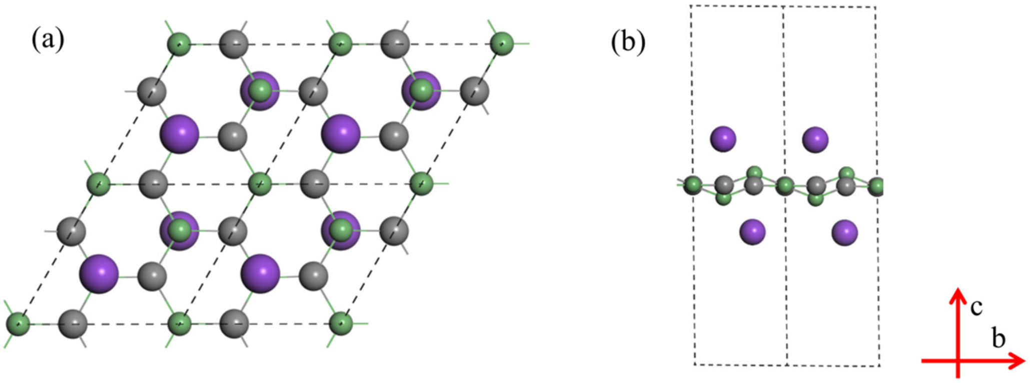

Figure S5. The (**a**) top view and (**b**) side view of stable K-adsorbed g-GeC structure in the way of two-surface adsorption in $\sqrt{3}\times\sqrt{3}\times1$ supercell with the ratio of K/C of x = 0.67

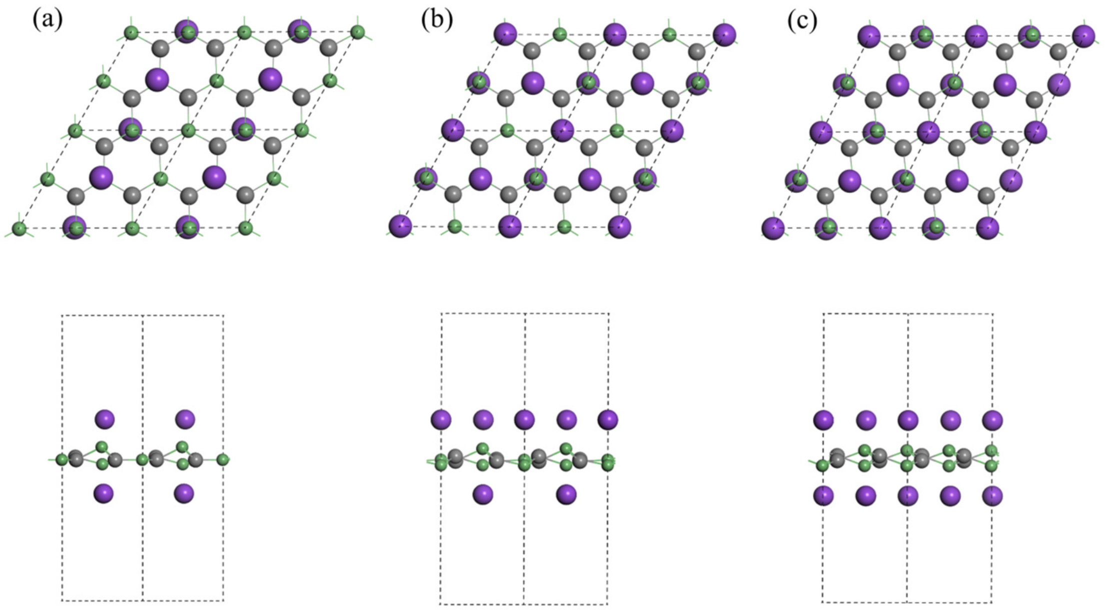

Figure S6. The top and side views of stable K-adsorbed g-GeC structure in the way of two-surface adsorption in 2 × 2 × 1 supercell with the ratio of K/C of (**a**)x= 0.5 , (**b**) x=0.75 and (**c**) x=1

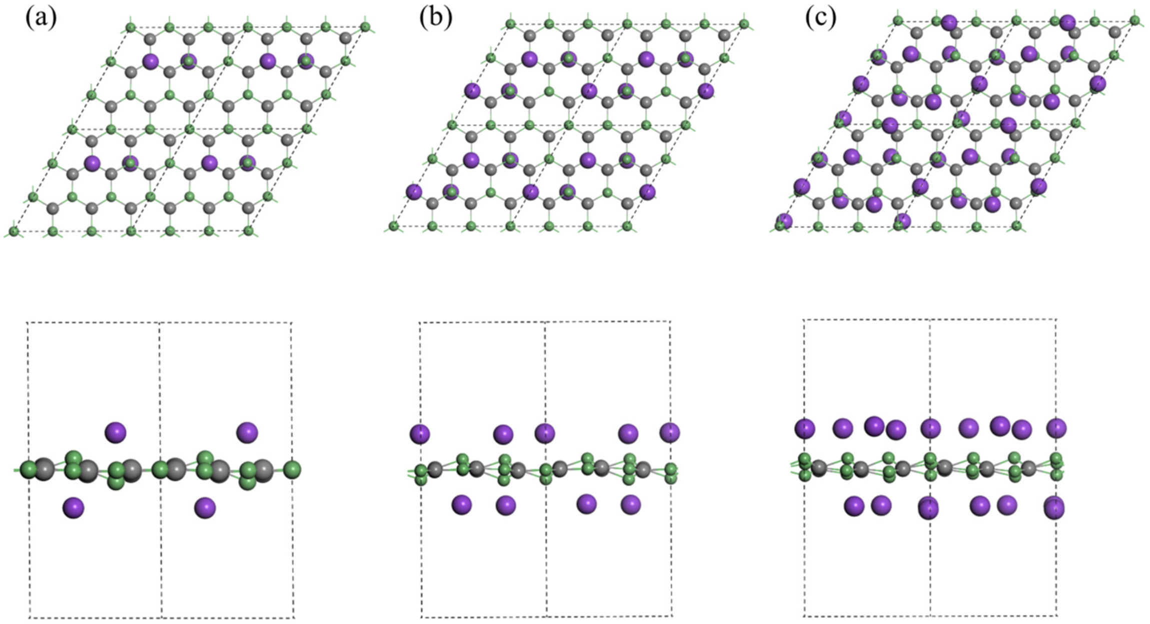

Figure S7. The top and side views of stable K-adsorbed g-GeC structure in the way of two-surface adsorption in 3 × 3 × 1 supercell with the ratio of K/C of (**a**) x=0.22, (**b**) x=0.44 and (**c**) x=0.89

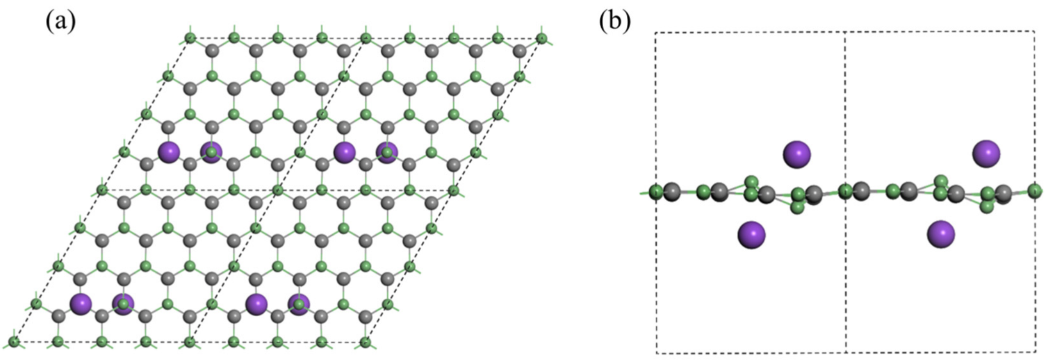

Figure S8. The (**a**) top view and (**b**) side view of stable K-adsorbed g-GeC structure in the way of two-surface adsorption in 4×4×1 supercell with the ratio of K/C of x=0.125

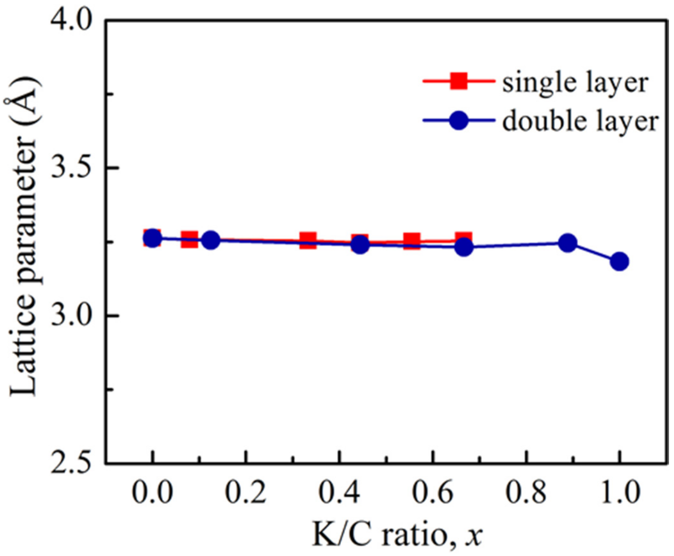

Figure S9. The lattice parameter for lattice constant *a* and *b* as functions of the ratio of K/C for the adsorption ways of single layer on one side of g-GeC and two sides of g-GeC (double layer model).

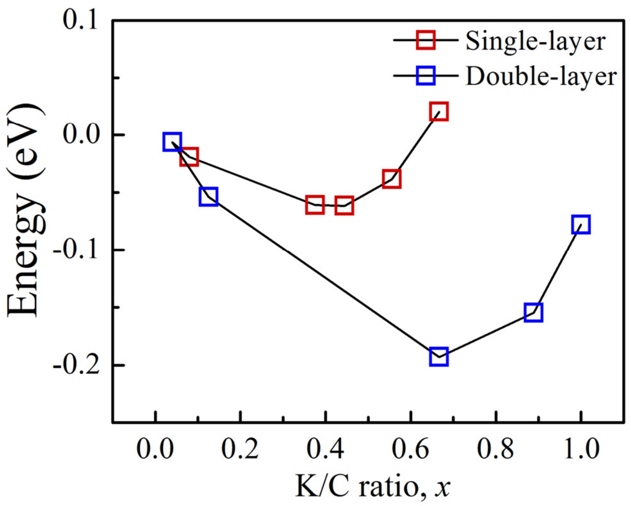

Figure S10. Formation energies as the functions of K concentration on g-GeC for the unilateral (single-layer model) and bilateral (double-layer model) adsorption.

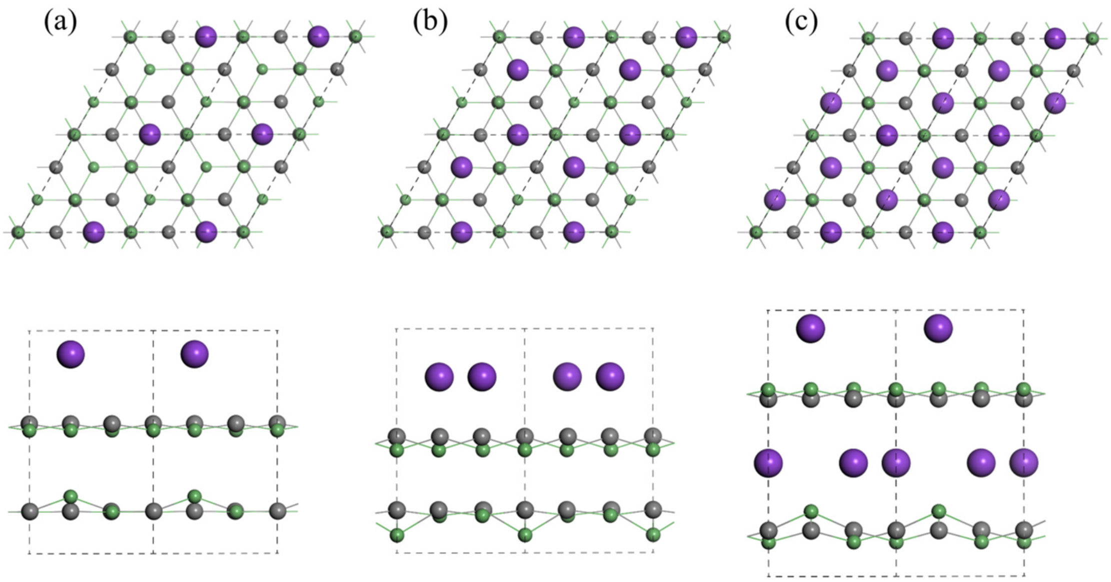

Figure S11. The top and side views of stable K-adsorbed bulk g-GeC structure in √3×√3×1 supercell with the ratio of K/C of (**a**) x=0.167, (**b**) x=0.33 and (**c**) x=0.5

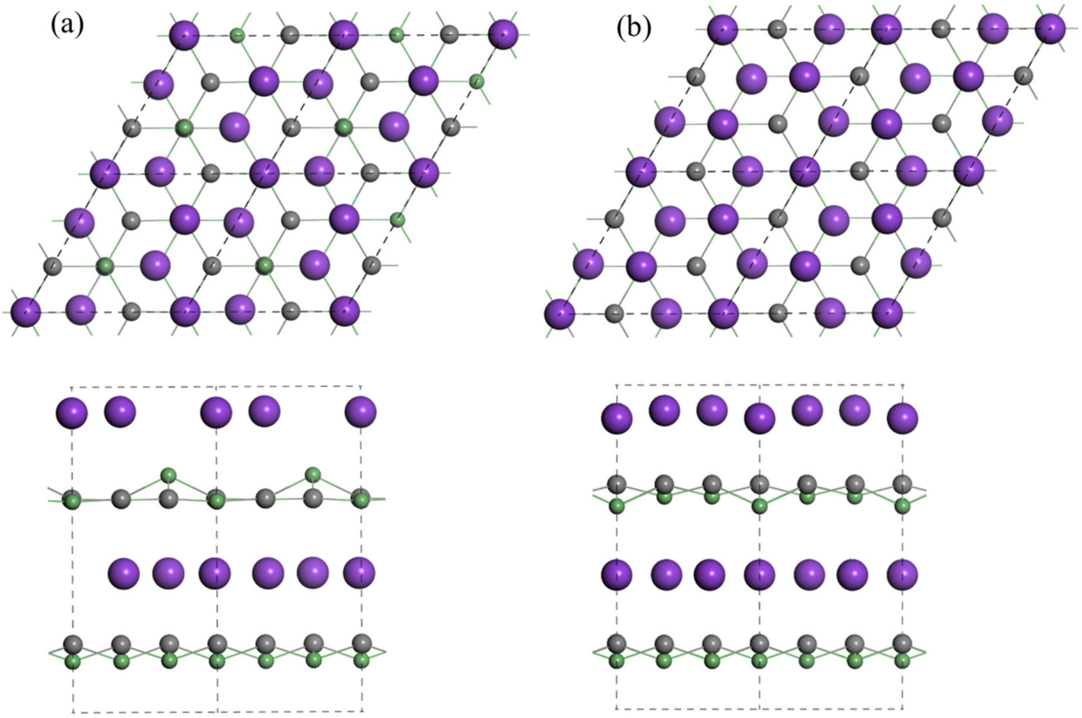

Figure S12. The top and side views of stable K-adsorbed bulk g-GeC structure in √3×√3×1 supercell with the ratio of K/C of (**a**) x=0.83 and (**b**) x=1

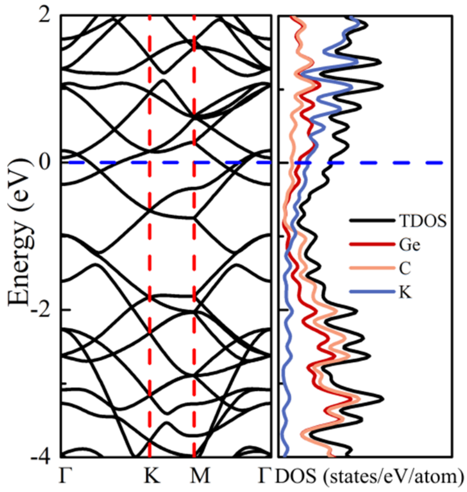

Figure S13. Band structure and partial density of states of K-adsorbed g-GeC in the way of one-surface adsorption in 3×3×1 supercell with the ratio of K/C of x=0.67
